# Update on Neonatal Isolated Hyperthyrotropinemia: A Systematic Review

**DOI:** 10.3389/fendo.2021.643307

**Published:** 2021-08-18

**Authors:** Ana E. Chiesa, Mariana L. Tellechea

**Affiliations:** Centro de Investigaciones Endocrinológicas “Dr. César Bergadá”, (CEDIE) CONICET – FEI – División de Endocrinología, Hospital de Niños Ricardo Gutiérrez, Buenos Aires, Argentina

**Keywords:** newborn screening, levothyroxine, subclinical hypothyroidism, systematic review, neonatal hyperthyrotropinemia

## Abstract

The purpose of this paper was to systematically summarize the published literature on neonatal isolated hyperthyrotropinemia (HTT), with a focus on prevalence, L-T4 management, re-evaluation of thyroid function during infancy or childhood, etiology including genetic variation, thyroid imaging tests, and developmental outcome. Electronic and manual searches were conducted for relevant publications, and a total of 46 articles were included in this systematic review. The overall prevalence of neonatal HTT was estimated at 0.06%. The occurrence of abnormal imaging tests was found to be higher in the persistent than in the transient condition. A continuous spectrum of thyroid impairment severity can occur because of genetic factors, environmental factors, or a combination of the two. Excessive or insufficient iodine levels were found in 46% and 16% of infants, respectively. Thirty-five different genetic variants have been found in three genes in 37 patients with neonatal HTT of different ethnic backgrounds extracted from studies with variable design. In general, genetic variants reported in the *TSHR* gene, the most auspicious candidate gene for HTT, may explain the phenotype of the patients. Many practitioners elect to treat infants with HTT to prevent any possible adverse developmental effects. Most patients with thyroid abnormalities and/or carrying monoallelic or biallelic genetic variants have received L-T4 treatment. For all those neonates on treatment with L-T4, it is essential to ensure follow-up until 2 or 3 years of age and to conduct medically supervised trial-off therapy when warranted. TSH levels were found to be elevated following cessation of therapy in 44% of children. Withdrawal of treatment was judged as unsuccessful, and medication was restarted, in 78% of cases. Finally, data extracted from nine studies showed that none of the 94 included patients proved to have a poor developmental outcome (0/94). Among subjects presenting with normal cognitive performance, 82% of cases have received L-T4 therapy. Until now, the precise neurodevelopmental risks posed by mild disease remain uncertain.

## Introduction

The state of mild elevated venous TSH (e.g., ≥6–20 mU/L) beyond 21 days of life with thyroid hormone concentrations within the normal range is termed or described as isolated hyperthyrotropinemia (HTT) or subclinical hypothyroidism (SCH) (1–3). The term SCH is more commonly used after infancy, when peripheral thyroid hormone levels are within the normal range but TSH is mildly elevated.

Before newborn screening (NBS) for congenital hypothyroidism (CH), most cases of mild CH and HTT remained unidentified due to their unapparent clinical course. Over a decade, the TSH cutoff value established by NBS programs has been adjusted downward, resulting in an increasing trend in the diagnosis of infants with mild CH and HTT in TSH-based NBS programs.

In general, it is difficult to obtain a true prevalence of the cases of neonatal isolated HTT for various reasons. First, certain studies do not report FT4 levels and confusion arises regarding the differentiation of mild CH from HTT, which might represent a continuum along a scale of thyroid dysfunction (4). Some studies do not distinguish between transient mild CH or borderline CH, and transient HTT, and describe all such cases as “transient TSH elevation” or transient hypothyroidism. Second, published studies have employed varying criteria for defining this condition: NBS programs have applied different TSH cutoff values, and studies have used initial (dried) whole blood spot TSH, first and second blood spot TSH levels, cord blood (dried or serum) TSH, or serum TSH (obtained at NBS or recall examination). Usually, the day of extraction is not the same for all NBS programs and the cutoff point is not adapted to the reality of time collection.

The criteria for assessing CH severity can be posed in terms of clinical, biochemical, and radiological features. Biochemically, CH severity can be judged as mild, moderate, or severe based on the serum FT4 concentration (<5, 5 to <10, and 10 to 15 pmol/L, respectively) (2). While in CH thyroid hormone levels are in general decreased, FT4 levels are, by definition, within the normal range in isolated HTT.

Neonatal isolated HTT can be transient or persistent. Transient neonatal HTT is usually defined as an abnormal transient elevation of neonatal serum TSH with normal T4 values. Transient neonatal HTT should be differentiated from false-positive NBS tests, defined as an abnormal screening test value, with normal results of serum tests taken immediately afterward (usually at 2 weeks of age). Most newborns suspected of CH because of elevated borderline neonatal TSH levels would have normal, or nearly normal, TSH, and normal FT4 after a few days, at the recall examination. Newborns with very short-lasting HTT should be classified as false-positive at NBS because the initial neonatal abnormalities can be attributed to a variety of transitory causes that have no significant adverse clinical consequences (5). Specimens collected in the first 24 to 48 h of life may lead to false-positive TSH elevations when using any screening test approach (1). During the first few hours of life, newborns experience a physiological increase in TSH levels in response to the environment, followed by a progressive decrease in its levels. The occurrence of high neonatal TSH (TSH ≥ 5 mU/L) decreases with the number of days from birth to sampling (6). It has been also demonstrated that the concentration of neonatal TSH decreases with age until it stabilizes at between 11 and 15 days of life (7).

Transient HTT has been mainly attributed to either iatrogenic iodine overload or iodine deficiency during fetal and early postnatal life (8). Transient mild CH and transient HTT are also usually proposed to be associated with maternal ingestion of goitrogenic substances which reach the fetus *via* placental transfer, or by maternal–fetal transfer of thyrotropin receptor (TSHR)-blocking antibodies, which are IgG immunoglobulins (8–10).

Neonatal HTT may also result from prematurity and/or low birth weight. TSH concentrations were found to be higher in small-for-gestational-age infants (11). A high incidence of HTT in premature neonates has also been reported, particularly in those small-for-gestational-age (12). Although the reason for the increased TSH levels is unknown, its origin could be multifactorial as many hypotheses have been proposed (11). TSH elevation in infants with Down syndrome is also highly prevalent during the neonatal period (3). A Turkey study has demonstrated HTT in 32 of the 80 newborns with Down syndrome (13).

Most reported cases of neonatal HTT occur as a transient form, while the persistent form is less well understood (14). Distinguishing between transient and persistent forms of HTT at the time of diagnosis is difficult and generally requires a large time frame (4). In general, the decisive consistent distinguishing factor between transitory and persistent forms of HTT is a reassessment of thyroid function at the time of treatment withdrawal, occurring in general after 2 or 3 years of age.

Finally, there is also controversy regarding the need for levothyroxine (L-T4) therapy in neonatal HTT (3). Many practitioners elect to “play safe” and treat infants with HTT to prevent any possible adverse developmental effects. Some studies have reported cases of mild CH or isolated HTT progressing to overt (more severe) hypothyroidism. As the developing brain has a critical dependence on thyroid hormone for the first 2 or 3 years of life, it is prudent to assure normal thyroid hormone levels during this period.

The purpose of this paper was to systematically review the published literature to compile—and, when possible, quantify—the existing data on neonatal HTT. Our aim has been primarily to address the following questions. First, how does the reported frequency of HTT vary depending on the mode of identification, and what other factors it depends on? Second, what is the most common cause of neonatal HTT? Third, what have we learned about management and clinical progression from follow-up and re-evaluation studies? Finally, is there any convincing evidence about the impact on cognitive development in these infants that supports L-T4 replacement therapy?

## Methods

The literature search was done on studies up to August 8, 2020, on the PubMed database from the National Library of Medicine using the following keywords and terms: (congenital OR neonatal OR newborn) AND (“subclinical hypothyroidism” OR “compensated hypothyroidism” OR hyperthyrotropinemia OR hyperthyreotropinemia OR hyperthyrotrophinaemia OR hyperthyrotropinaemia). Searches were limited to studies on humans that were published in English. Eligible studies had no minimum number of participants and there were no country restrictions. All the studies that investigated the prevalence, L-T4 management, re-evaluation of thyroid function during infancy or childhood, etiology including genetic variation, thyroid imaging test results, bone maturation, and/or developmental outcome were considered in this study. Data on L-T4 therapy were carefully extracted from each study when available. Articles were screened using the following inclusion criteria: 1) must contain information on neonatal HTT, preferably with data on TSH and FT4 levels, and a clear phenotype can be inferred (it is possible to discriminate HTT from CH, especially from mild CH); and 2) HTT must have been diagnosed during the neonatal period or early infancy (1–3 months of age). For large cohorts, included subjects should have less than 1 year of age at diagnosis and on average no more than 3 months of age. Patients with increased serum TSH levels, evaluated at recall examination because of a positive NBS or for other reasons (e.g., a first-degree relative of an index case), were included.

A formal variant classification was assigned according to the recommendations from the American College of Medical Genetics (ACMG). The variant interpretation was performed with Varsome (hg19) (15). Variants labeled as uncertain significance (VUS) according to Varsome were further revised and reclassified if necessary, according to functional studies and evidence from Variant Effect Predictor (VEP, Ensembl).

Cases presenting with mild CH or apparent low levels of T4/FT4 were excluded. Cases positive at NBS with normal serum TSH at reexamination were excluded. Studies were also excluded when it was not possible to identify if participants had increased serum TSH at confirmation of diagnosis (exceptions were cases receiving L-T4 treatment, since it may be inferred that TSH levels were confirmed in serum). Excluded were also studies conducted on cohorts with maternal or neonatal disease (except for thyroid disease) or genetic syndromes (except for resistance to TSH); studies conducted entirely on premature, small-for-gestational-age, low birth weight, or Down syndrome subjects; studies reporting exclusively synonymous single nucleotide variants (SNVs) and common sequence variants (“genetic polymorphisms”); and review articles and studies reporting only *in vitro* experiments.

## Results

### Literature Search and Case Inclusion

**Figure 1:**
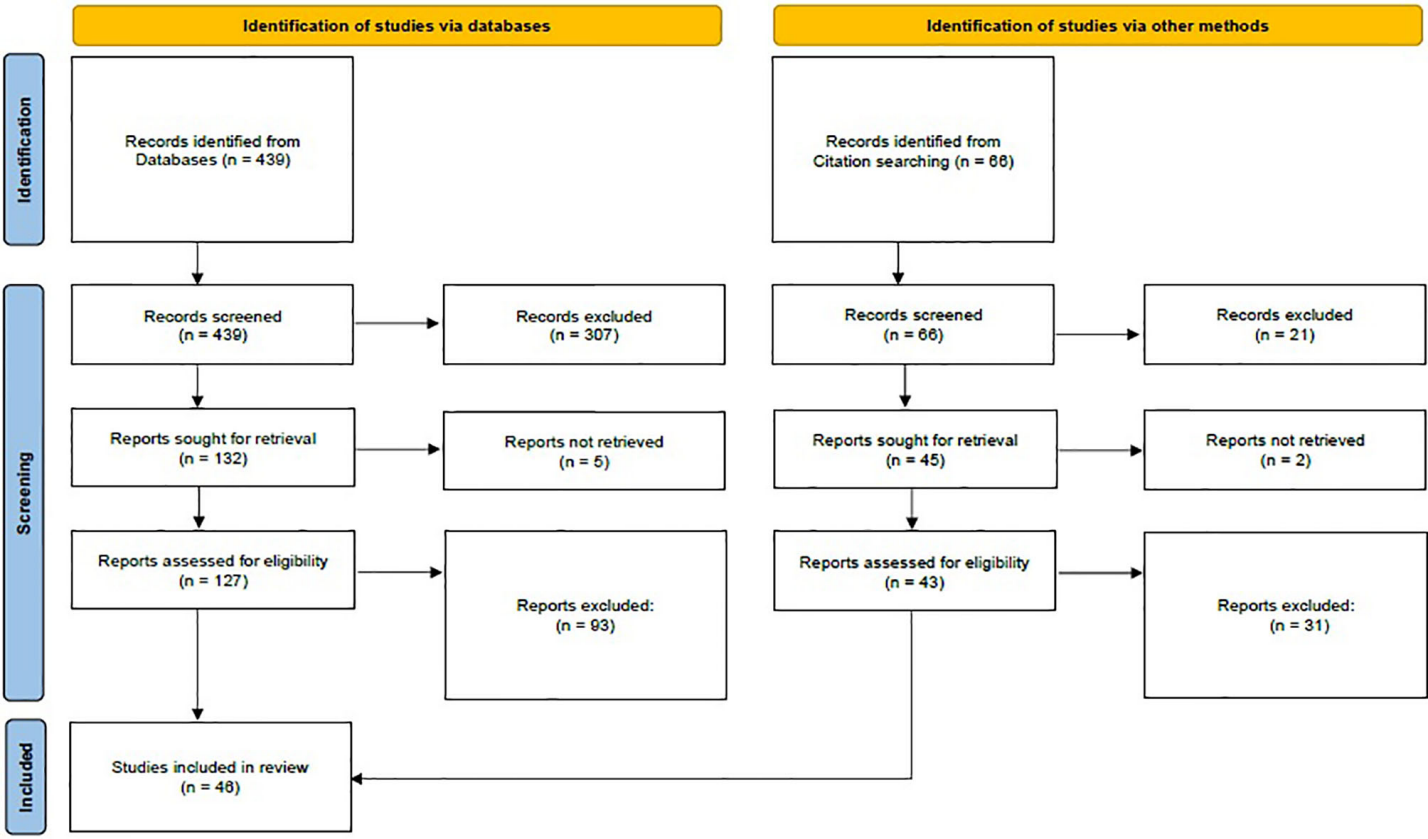
PRISMA flow diagram. Adapted from Page et al. (30).

The PubMed search strategy resulted in 439 hits. Reviews and experimental papers were used to perform a further search, which revealed an additional 66 records. The initial screen was based on title, abstract, and occasional whole-text scan. After the in-depth screening, 170 relevant citations remained for further review. All these articles were thoroughly read and evaluated. Eligible studies were independently reviewed by two reviewers; 109 were consistently excluded by both authors, while 15 additional studies were excluded after discussion and agreement between reviewers. Nine studies including subjects positive at NBS with normal serum TSH at reexamination were excluded (**Table S1**). In those studies, reexamination was performed beyond 2 weeks of life (range 10–90 days), and therefore, it is not possible to address if serum TSH was elevated at 14 days of life and became normal shortly afterward. One study was excluded because of wide inclusion criteria, and five studies were excluded since increased TSH values detected at NBS were not further confirmed by serum analysis (**Table S1**). Finally, 46 citations were used to build the nine summary tables included in this systematic review (**Tables 1**, **2** and **Tables S2–S8**). A flowchart of the article selection process is shown in **Figure 1**.

**Table 1:** Prevalence of neonatal hyperthyrotropinemia.

Id	Country	Period	Condition	Description	Prevalence (%)	HTT : CH	Patients receiving L-T4
Fu C. 2017 (16)	China	2009–2016	HTT	Increased TSH (>10 mU/L) and normal FT4 at recall (days 7–28)	911/1,238,340 (0.07)	911:731	0/911
Kumorowicz-Czoch M. 2011 (17)	Poland	2000–2006	HTT	Increased TSH (>9.1 mU/L) and normal FT4 at recall (day ~26)	4/233,120 (0.002)	4:58	4/4, trial-off at ~4 years
Corbetta C. 2009 (18)	Italy	1999–2005	HTT	Mildly increased TSH (5.0–9.9 mU/L) and normal/high FT4 at recall	578/629,042 (0.09)	578:435	0/578
Nishiyama S. 2004 (9)	Japan	2000–2002	HTT	Mildly increased TSH (cutoff value na) and normal serum FT4 at recall	24/37,724 (0.06)	24:6	14/24
Tyfield L.A. 1991 (19)	England	1981–1987	HTT	Increased TSH (>10 mU/L) and normal T4 at recall (day ~30)	3/185,723 (0.002)	3:45	0/3
Miki K. 1989 (20)	Japan	1975–1983	t-HTT	Increased TSH (>17 mU/L) at recall (2–8 wk) but normal at 2–9 mo, TH in the normal range, + other criteria	16/281,468 (0.006)	na	5/16, follow-up
Sava L. 1984 (21)	Italy	(30 mo)	t-HTT	Increased TSH (>8 mU/L) and normal T4 at recall (day ~32), but normal TSH at wk 3–6	11/7,953 (0.1)	11:4	0/11
Czernichow P. 1981 (22)	Belgium	1979–1980	t-HTT	Increased TSH (>40 mU/L) and normal TH at recall (days 15–30), TSH decreases to normal after 5 mo	3/10,261 (0.03)	na	0/3
Miyai K. 1979 (23)	Japan	1975–1978	t-HTT	Increased TSH (>12 mU/L) and normal TH at 2 mo, TSH decreases to normal after 7–9 mo	1/91,400 (0.001)	1:9	0/1
Summary estimates					1,551/2,715,031 (0.06)	1,532:1,288	23/1,551 (1.5%)

t-HTT, transient HTT; TH, thyroid hormones; mo, months; wk, weeks; na, not available.

**Table 2:** Developmental outcome in neonatal hyperthyrotropinemia.

Id	Condition	Observations	*N* cases	L-T4	Test and/or assessment	Impaired developmental outcome	Age (years)*
Rovelli R. 2010 (24)	HTT	No preterm, asphyxia, or congenital disease	3	3/3	Mental development (Griffith’s scale)	0/3	1–2
Demirel F. 2007 (25)	HTT	na	36	36/36	Denver developmental screening test	0/36	3
de Roux N. 1996 (26)	HTT	1/4 preterm	4	3/4	Intellectual development	0/4	na
Nishiyama S. 2004 (9)	HTT	Full-term	15	12/15	Psychomotor development	0/15	2
Tomita Y. 2003 (27)	p-HTT	GA 36–40 wk, BW 2,090–3,580 g	14	14/14	Japanese Denver developmental screening test	1/14 (DS)	~3–6
Vigone M.C. 2005 (28)	p-HTT	na	1	1/1	Mental development (Griffith’s scale)	0/1	4
Mizuno H. 2009 (29)	p-HTT	Full-term, BW 2,374–3,450 g	4	4/4	Intelligence quotient	0/4	6
Tyfield L.A. 1991 (19)	p-HTT	Uneventful pregnancy and neonatal period	3	0/3	Developmental status regarded by parents and pediatricians	0/3	5–6
Miki K. 1989 (20)	t-HTT	Full-term, normal BW	16	5/16	Psychomotor development (Tsumori and Isobe scale) or intelligence (Wechsler scale)	1**/16 (deafness)	2–7
Summary estimates			96	–	–	2/96	1–7

Data in column “L-T4” are expressed as a ratio of the number of LT4-treated patients to the total number of cases. Data in column “Impaired developmental outcome” are expressed as a ratio of the number of participants with “abnormal” results to the total number of cases.

p-HTT, persistent HTT; t-HTT, transient HTT; GA, gestational age; BW, birth weight; DS, Down syndrome; wk, weeks; na, not available.

*Age at assessment.

**Untreated patient (Miki K. 1989).

### The Changing Prevalence of Neonatal HTT

Nine studies reported on the prevalence of HTT in a total of 2,715,031 infants. The overall prevalence of HTT was estimated at 0.06% (1,551/2,715,031), but prevalence varied widely among studies (range 0.001%–0.1%, **Table 1**). The estimated prevalence of HTT in both Europe and East Asia is also 0.06% (**Table 1**). Finally, the computed HTT: CH ratio is 1.2:1 (1,532:1,288; data available from seven studies).

### Clues to the Etiology of Transient HTT

We examined the potential causes of transient HTT such as either iodine overload or iodine deficiency, maternal ingestion of goitrogenic substances, and maternal–fetal transfer of TSHR-blocking antibodies.

Six studies comprising 77 subjects evaluated serum and/or urinary iodine concentration in neonatal HTT (**Table S2**). Cutoff values for determining abnormal iodine concentrations were not available in some studies; 46% (19/41) and 16% (8/50) of infants had increased and decreased iodine levels, respectively.

Placental transfer of maternal IgG antibodies against the thyroid TSHR is another putative cause of transient neonatal HTT. We found only five studies investigating the presence of anti-TSHR antibodies (TRAbs) and/or TSHR stimulatory antibodies in the mother and/or in the infant (**Table S3**). Information about maternal thyroid disease was also collected. Two studies have demonstrated the presence of TRAbs in cases of neonatal HTT (Azzopardi P 2010), or in the mothers of those infants (Evans C 2011), suggesting transplacental transfer of maternal antibodies (31, 32). Two additional studies have also reported transient HTT due to maternal autoimmune thyroid disease with the presence of anti-TSHR antibody activity in both maternal and infant serum (Tamaki H 1989, Schwingshandl J 1993) (33, 34).

No studies reporting on the association between neonatal HTT and maternal ingestion of goitrogenic substances were found in this systematic review of the literature.

### Thyroid Imaging in Neonatal HTT

**Figure 2:**
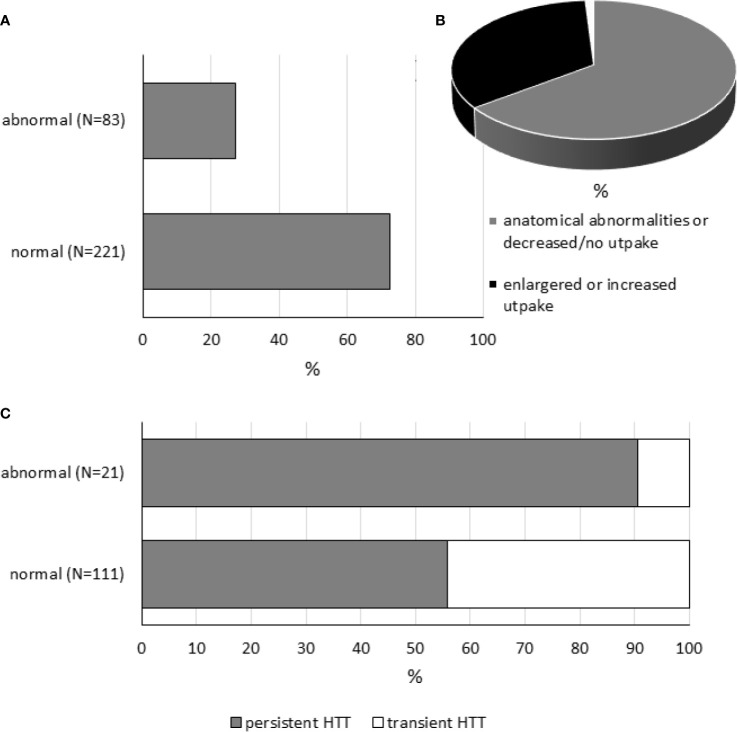
Thyroid imaging tests in neonatal HTT. Panel **(A)** shows the percentages of cases with normal and abnormal findings. Panel **(B)** is a pie chart showing the proportion of patients with anatomical abnormalities (hypoplasia, hemiagenesis, or ectopic thyroid gland) or decreased radionuclide uptake or no uptake (in gray, *N* = 54), enlarged thyroid gland, or increased radionuclide uptake (in black, *N* = 28), or data not available (in white, *N* = 1). Panel **(C)** shows the percentages of persistent and transient conditions in subjects with normal or abnormal findings.

Newborns with isolated HTT may have mild changes in thyroid morphology and/or genetic abnormalities. **Table S4** and **Figure 2** compile data of 304 subjects extracted from 28 included studies (33 datasets) reporting thyroid imaging results. Thyroid morphology and/or function was more frequently assessed by ultrasonography and Technetium-99m (99m-Tc) scintigraphy and less commonly by radioiodine scan; 27% (83/304) of included subjects showed thyroid gland abnormalities. Of 83 subjects with abnormal results, 34% (28/83) showed enlarged thyroid gland or increased radionuclide uptake, while 65% (54/83) subjects showed anatomical abnormalities (hypoplasia, hemiagenesis, or ectopy), decreased radionuclide uptake, or no uptake.

As expected, the occurrence of thyroid abnormalities was found to be higher in the persistent form of HTT (persistent: 19/81, 23% *vs.* transient: 2/51, 4%, **Table S4**); however, it should be also considered that persistent forms are more likely to be examined by thyroid imaging than transient conditions. Note that, for some datasets, detailed information related to conditions was not available (**Table S4**). On the other hand, among subjects with abnormal thyroid imaging tests, different frequencies of persistent and transient forms of neonatal HTT were found (persistent: 19/21 *vs.* transient: 2/21, **Figure 2**).

The number of patients with abnormal imaging tests receiving L-T4 therapy was estimated from datasets where information about L-T4 treatment was available. Interestingly, between those subjects with abnormal thyroid imaging tests, 77 of 88 (87.5%) patients have received L-T4 therapy (one dataset was excluded from computation because treatment information was not available, see **Table S4**).

Finally, the study of Nishiyama S 2004 (9) was included in this systematic review but not incorporated into **Table S4** for the reason that it is not possible to obtain the exact number of subjects having abnormal imaging tests. Thyroid volumes measured using ultrasonography were found to be increased with respect to normal controls in 15 infants with neonatal HTT.

### Insights Into Genetic Variation in Neonatal HTT

**Table S5** compiles 37 cases extracted from 18 studies reporting genetic variants in neonatal HTT. Data were carefully extracted from studies with variable designs. Information about thyroid imaging tests, phenotype later in life, family background (first-degree relatives presenting with increased TSH levels), ethnicity, consanguinity, and study design was collected.

To date, genetic variation has been found in three genes in patients with isolated neonatal HTT (**Table S5**): *TSHR*, thyroperoxidase (*TPO*), and dual oxidase 2 (*DUOX2*). Overall, 35 different genetic variants have been found in 37 cases (**Table S5**).

Cases were found to be heterozygous, homozygous, or compound heterozygous. Biallelic carriers were more frequently reported than monoallelic (heterozygous) carriers (25 *vs.* 12 cases). Intriguingly, an Israeli group has reported a case of neonatal isolated HTT who required L-T4 therapy carrying two heterozygous missense variants in the *TSHR* gene and one heterozygous missense variant in the *TPO* gene (35).

**Figure 3:**
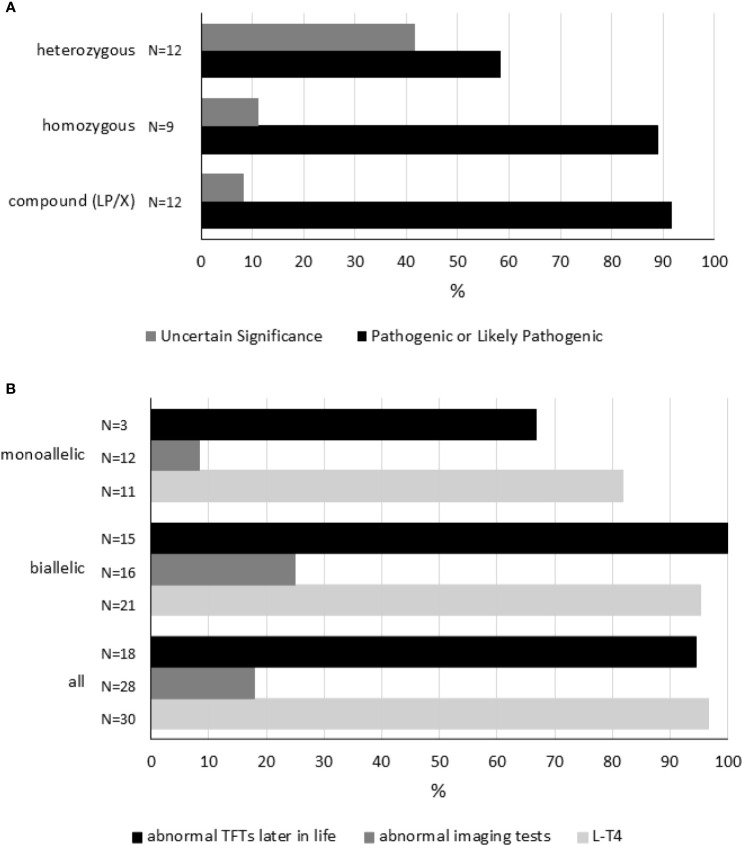
Genetic variation in the *TSHR* gene. Panel **(A)** shows each reported genotype [compound heterozygous (LP/X), homozygous and heterozygous] representing a cluster. As disease severity will depend on the combination of variants in every locus, variants were interpreted and classified (see **Table S6**). Within each cluster, the percentages of each combination of sequence variants are shown. “Pathogenic” and “Likely Pathogenic” were grouped together. Panel **(B)** shows 1) the percentage of subjects receiving L-T4 therapy, 2) the percentage of cases showing abnormal imaging tests, and 3) the percentage of individuals having abnormal TFTs later in life according to genotype. Compound heterozygous (LP/X) and homozygous genotypes were grouped together as biallelic. TFTs, thyroid-function tests. For details, please see **Table S5**.

Until now, 26 different *TSHR* sequence variants have been documented in 33 cases with neonatal-onset HTT (**Table S5**). Twenty-five SNVs, both missense and nonsense, as well as a deletion (frameshift mutation) in the coding sequence, have been identified in 33 patients of different ethnic backgrounds (**Table S5**). **Table S6** shows the interpretation of the *TSHR* sequence variants. Of those 25 SNVs, 20 were interpreted as pathogenic or likely pathogenic. T655*(delAC) causes the formation of a nonsense codon leading to the production of a receptor that lacks the entire transmembrane domain 7 and the intracellular (C-terminal) tail. According to the variant interpretation from **Table S6**, it can be speculated that in almost all patients the phenotype may be explained by the genotype. More specifically, 27 of 33 (82%) patients were carriers of (mono or biallelic) pathogenic (or “likely pathogenic”) variants in the *TSHR* gene (**Figure 3**). In six cases (see **Table S4**), homozygous or heterozygous variants of uncertain significance could not account for pathogenicity.

Homozygous *TSHR* variants were found in nine cases, six Japanese patients carrying R450H, two members of consanguineous kindreds, and one subject born to consanguineous parents (**Table S5**).

Among all *TSHR* variants, R450H was most frequently reported. R450H was observed in Japan in 12 cases (6 compound heterozygous and 6 homozygous cases, **Table S5**).

Variants are distributed throughout the *TSHR* gene with no evident hot spot. As shown in **Table S6**, 54% of the reported sequence variants are distributed in the extracellular domain.

Although the number of studies reporting genetic studies is scarce, some conclusions can be drawn about the characteristics of subjects carrying *TSHR* sequence variants (**Table S5** and **Figure 3**). First, L-T4 therapy is frequently initiated during the neonatal period in cases carrying monoallelic or biallelic variants. More specifically, L-T4 therapy was started in 97% of cases (29/33, in 3 cases information related to L-T4 treatment was not available). Second, thyroid imaging may not be correlated to genetic variation. Abnormal imaging tests were found only in 5 of 28 (18%) cases carrying sequence variants in the *TSHR* gene (in 5 cases information about thyroid imaging tests was missing). And third, mildly altered thyroid tests (slightly increased TSH and/or abnormal TRH test) are commonly (94%, 17/18) observed during childhood or adolescence in these patients (in 15 cases information was not available).

Genetic variation in *TPO* and *DUOX2*, key genes required for thyroid hormone synthesis, has been reported in neonatal HTT (**Table S5**). Kotani and colleagues described a partial iodide organification defect caused by likely pathogenic sequence variants in the *TPO* gene (36). *DUOX2* pathogenic sequence variants may also cause mild to moderate forms of hypothyroidism, including transient CH and transient HTT (37). In this systematic review, six different *DUOX2* (missense, nonsense, and frameshift) variants were found in infants with neonatal-onset HTT without goiter, consistent with resistance to TSH, rather than dyshormonogenesis (**Table S5**).

### Management: To Treat or Not to Treat?

**Table S7.1** compiles 10 follow-up studies (including 476 subjects) where some or all participants were treated with L-T4 and re-evaluation of thyroid function was performed after withdrawal of treatment, usually at ~2–3 years of age. Information about L-T4 discontinuation (before trial-off medication) to prevent overtreatment and progression to overt hypothyroidism was collected when available. Data on thyroid status at re-evaluation and attempts of L-T4 withdrawal were also extracted, including information on successful and unsuccessful attempts.

More than half of the participants (60.5%, 288/476) were treated with L-T4 (**Table S7.1**). In general, treatment is started in infants with persistent mild TSH elevation and/or abnormal TRH test results.

During treatment, certain patients experienced elevated levels of thyroxine. In some cases, therapy was stopped, and patients showed full recovery within the first year of life. Levothyroxine cessation before trial-off was reported in four studies, with an estimated overall percentage of L-T4 discontinuation of 11% (32/281, **Table S7.1**).

After the patient reached 2–3 years of age (range 1–6 years), L-T4 treatment was ceased to conduct thyroid function tests. In those cases where TSH levels were within the normal range, patients were followed up without L-T4 therapy. However, according to **Table S7.1**, TSH levels were found to be elevated following cessation of therapy in 101 of 229 cases (44%).

In general, all patients had a confirmatory trial-off levothyroxine; however, in certain infants, discontinuation was never tried, especially in patients having elevated TSH levels while receiving therapy. Overall, therapy withdrawal was not attempted in 17.5% of cases (40/229, **Table S7.1**).

A matter of debate is whether to reassume L-T4 treatment once blood TSH is mildly elevated following the withdrawal of treatment in childhood. There remains a role for clinical judgment in the management of these cases. A persistently abnormal TSH was not always used as a rule of thumb to resume medication. It has been reported cases of increased TSH but a normal response to TRH test where treatment was not restarted (Demirel F 2007) (25), and cases of increased TSH where patients were followed until normal TSH concentration was reestablished (Tomita Y 2003) (27). Withdrawal of treatment was judged as unsuccessful, and medication was restarted, in 78% of cases (60/77, 7 datasets).

**Figure 4:**
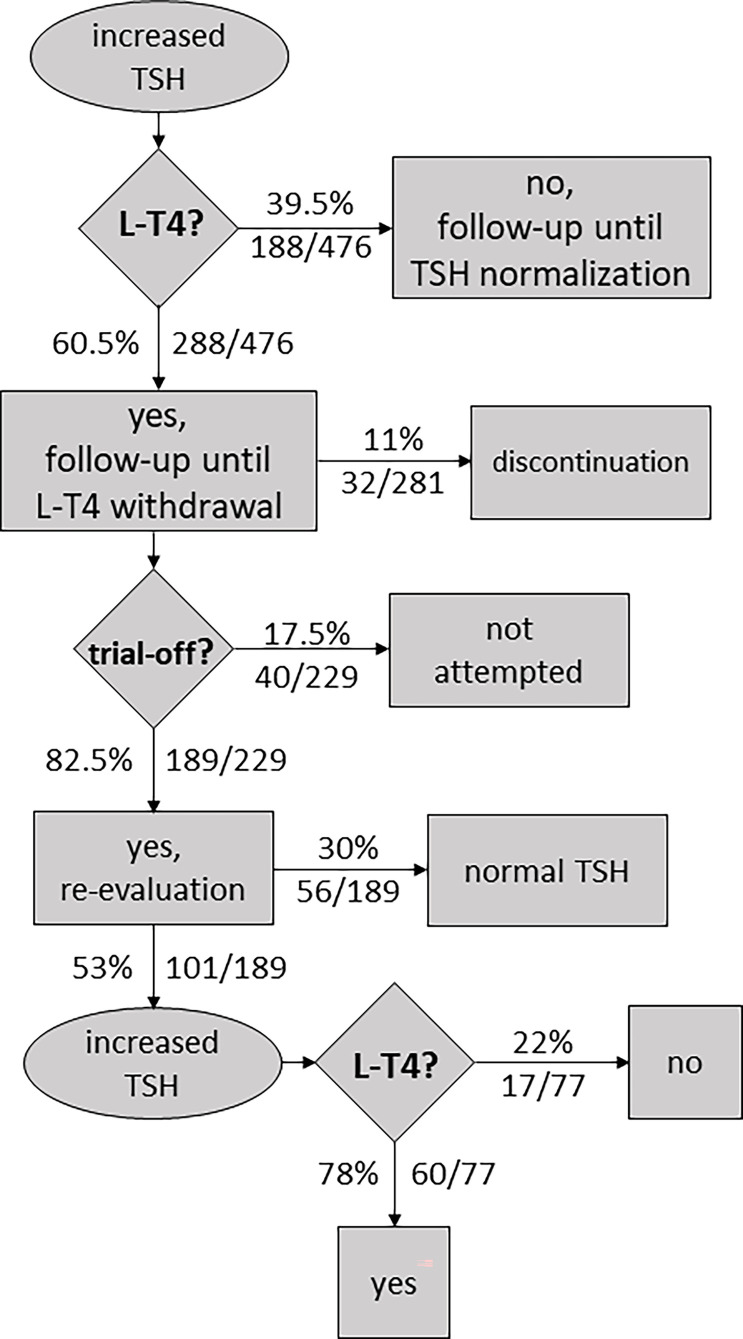
Flowchart illustrating an approach to the management of neonatal hyperthyrotropinemia based on the reviewed literature.

**Figure 4** shows a flowchart illustrating the approach to the management of neonatal HTT based on the reviewed literature.

### Clinical Evolution in Children With Neonatal-Onset HTT

Different cohort studies have been conducted to assess thyroid function progression in neonatal isolated HTT; however, most published studies are retrospective with low sample sizes. Overall, data collected from 14 studies (**Tables S6.1, 6.2**) indicate that the percentage of patients with persistent elevation of TSH levels during early childhood is not negligible (45%, 125/279).

### On the Topic of Growth Rate and Cognitive Development

Some studies have addressed the question of growth rate and cognitive development in children diagnosed during the neonatal period with HTT. In general, normal bone maturation was reported in all children. Data collected from nine studies showed that 2 of 74 (3%) cases with neonatal-onset HTT showed delayed bone age or ossification (**Table S8**). In contrast, evidence on an association between neonatal HTT and cognitive development is scarce and contradictory. We have compiled studies evaluating cognitive development. Data about perinatal outcomes (prematurity, birth weight, congenital disease, and related information) were extracted when available. Data collected from nine studies have shown that 2 out of 96 (2%) cases had abnormal cognitive outcomes during infancy or childhood (**Table 2**). However, after excluding two cases where the impairment of cognitive development would be related to congenital defects (deafness, Down syndrome), none of the remaining 94 subjects showed adverse developmental outcomes (0/94). Among subjects with normal cognitive performance, 82% (77/94) of cases received L-T4 therapy. Results should however be interpreted with caution since some studies have not assessed cognitive and/or motor abilities throughout standardized scales, and detailed information about diagnostic tools was not always available (**Table 2**).

## Discussion

The purpose of this paper was to systematically review the published literature to compile—and, when possible, quantify—the existing data on neonatal HTT. We have collected evidence extracted from 46 research articles in nine tables (**Tables 1**, **2** and **Tables S2–S8**). We aimed to address specific questions which are discussed below.

### How Does the Reported Frequency of HTT Vary Depending on the Mode of Identification, and What Other Factors Does It Depend on?

It has been demonstrated that several factors may affect the concentration of TSH, including maternal thyroid diseases and drugs, perinatal outcomes (including the type of delivery and birth conditions), and the methods and timing of TSH determination (38). Gender is a known factor influencing neonatal TSH level. The percentage of neonatal HTT is higher among male newborns, in contrast with the higher number of female newborns with CH in general (6, 16, 39, 40).

A proportion of transiently raised TSH may be attributable to analytical assay difficulties. A “maternal factor”, by means of maternal IgG antibodies, has been repeatedly reported to interfere in some old radioimmunoassays of TSH (22, 41–45). There is also some variation in the prevalence between geographical areas and countries. The disparity in the reported prevalence may also be in part to population genetics and ethnicity. The risk of high neonatal isolated TSH has been previously shown to be dependent on maternal ethnicity (6). A cohort study conducted in Skopje, the capital of Macedonia, also reported ethnic differences in the incidence of high serum TSH levels in neonates (46). Probably, an underestimation of the prevalence of HTT may occur in countries where the thyroid NBS is practiced by measuring the total T4 level, which is expected to be within the normal range in HTT (8). Finally, there is some discussion about adapting the threshold according to the time of year (6). Seasonal aggregation has been described for recall rates in Iran, with recall occurring significantly more in winter than in other seasons (47, 48).

Because neonatal HTT is identified on the basis of the amount of a hormone that has a continuous distribution in the general population, prevalence estimates certainly should scale as the cutoff is decreased (49). Within an individual NBS program, undoubtedly, an increase in recall rates and prevalence of HTT is expected by lowering cutoffs. Through a systematic review of the literature, we attempted to understand the nature of the variation in the reported frequency of HTT. Given that definitions and diagnostic criteria vary widely among NBS programs and studies, there are significant differences between the reported incidence rates of this condition, beyond the expected variation associated with different cutoff values (**Table 1**).

Certainly, there is still some doubt about the value of detecting cases of neonatal HTT through NBS programs. Lain and collaborators have outlined the arguments both for and against lowering TSH cutoffs at NBS, along with a section focused on the economic implications (50). The whole purpose of population-based biochemical screening is to identify medically actionable conditions, affected by treatment at a presymptomatic stage (51). While overt CH meets this goal, the evidence for neonatal HTT is vague, and whether mild abnormalities in thyroid function in the newborn have an impact on cognitive development remains controversial. Ultimately, NBS programs need to balance the goal of detecting such newborns versus the economic cost burden and parental anxiety. Accordingly, differing viewpoints are expected from a public health perspective and from the clinical pediatric endocrinologist.

Some actions can be taken to decrease the reexamination rate and the incidence of transient HTT. Iodine excess in babies is considered to be a factor that causes increases in recall rates and in the frequency of transient HTT, ultimately increasing screening costs (52). In this systematic review, excessive iodine levels were more frequently observed than insufficiency, and in general, maternal iodine availability acted as a disease modifier. Excessive and insufficient iodine levels were found in 46% and 16% of infants, respectively (**Table S2**). The fetus and newborn can be exposed to high maternal iodine concentrations either by crossing the placenta perinatally or by secretion of iodine into breast milk postnatally (9). To avoid excess consumption of iodine during gestation and lactation, pregnant women should be checked and advised for their iodine intake. Direct iodine overload in the newborn may be also caused by either disinfectant agents or contrast medium from practices performed during the perinatal period (9). The use of compounds without iodine for antisepsis should be recommended in the peripartum period (52). On the other hand, iodine insufficiency in pregnancy would be avoided with iodine supplements or iodized salt. Deficient maternal iodine values are associated with high TSH values. It has been demonstrated that women who give birth to infants with false-positive results at NBS have lower iodine concentrations as compared with mothers who give birth to infants with normal cord blood TSH levels (53).

Among the etiologies for transient HTT, TSHR-blocking antibodies seem not to significantly increase the incidence rate. Different generations of competitive binding TRAb tests for the detection of TSHR-binding inhibitory immunoglobulin have been used in the serological diagnosis of thyroid disease, principally in Graves’ disease. Of note, the TSHR stimulatory antibodies can only be detected by cell-based bioassays and should be differentiated from the TSHR-blocking antibodies (34). The presence of TSHR-blocking antibodies is investigated by the measurement of TRAbs or thyrotrophin-binding inhibitor immunoglobulins. On the other hand, *in vitro* assays can demonstrate the presence of thyroid-stimulation-blocking antibodies, serum immunoglobulins that had the ability to inhibit thyroid adenylate cyclase stimulation. The diagnosis of transient HTT should be suspected especially if the mother has autoimmune thyroid disease. In fact, a higher recall rate in NBS in infants of mothers with hypothyroidism and autoimmune thyroiditis has been reported, and most of them returned to normal during follow-up (45, 54). A family history of neonatal high thyrotropin with normal thyroid function which resolves spontaneously deserves the measurement of TSHR antibodies. Although TSHR-blocking antibodies are a rare cause of transient HTT, autoimmune thyroid disease is quite prevalent in the general population.

Finally, exposure to antithyroid drugs would substantially contribute to the incidence rate of transient HTT, given the high prevalence of maternal hyperthyroidism; however, no studies reporting on the association between neonatal HTT and maternal ingestion of goitrogenic substances were found.

Identifying the mechanisms behind transient HTT would make it possible to discriminate at an early stage between the transient and the persistent forms and, thus, reduce medication and follow-up expenses.

### Is Genetic Variation the Most Common Causation or Origination of Persistent Neonatal HTT?

A continuous spectrum of thyroid impairment severity can occur because of either genetic or environmental factors, or both (5).

In this systematic review, 35 different genetic variants have been found in three candidate genes (**Table S5**). In the 37 included cases, biallelic carriers are more frequently described than monoallelic carriers of genetic variants.

Resistance to TSH is a genetic disease characterized by hyposensitivity to a biologically active TSH molecule. This definition excludes autoimmunity with TSHR-blocking antibodies mimicking the phenotype of resistance to TSH (55, 56). The phenotype is determined by the degree to which the function of the “mutant” TSH receptor is diminished (29). Affected individuals have elevated serum TSH in the absence of goiter, with the severity ranging from isolated HTT to severe CH (55). As expected, resistance to TSH is commonly caused by sequence variants in the *TSHR* gene (56).

Until now, 26 different *TSHR* sequence variants have been documented in 33 cases with neonatal-onset HTT (**Table S5**), half of them distributed in the extracellular domain (**Table S6**). The human *TSHR* gene encodes for a G-protein-coupled receptor with a classical seven-transmembrane domain interacting with G proteins and an extracellular domain. The TSHR extracellular domain is unusually large and encoded by exons 1 to 9 and part of exon 10 (both transmembrane and intracellular domains are encoded entirely by exon 10). Eighty-two percent of the cases were found to be carriers of pathogenic (or “likely pathogenic”) variants in the *TSHR* gene (**Tables S5, S6**), and thus, genetic variants may explain the phenotype of the patients.

Different researchers have tried to elucidate the genetic variation that may be involved in the etiology of isolated neonatal HTT. Calaciura et al. have examined a cohort of children with neonatal HTT or false-positive results at NBS and found that 3 of 45 children were carriers of heterozygous variants in two candidate genes, *TPO* and *TSHR* (57). In another study, simple or compound *TSHR* heterozygous variants were detected in up to 30% (34/111) of children with SCH, half of whom were positive at NBS (17/34), with a high prevalence of first-degree family history for SCH (58).

Of note, some published studies were not able to detect genetic variation in candidate genes in cases with neonatal-onset HTT (10, 59–61). Worth mentioning, neonatal HTT may also be found in the context of complex syndromes, like pseudohypoparathyroidism (8, 62–64). Nowadays, there is no estimate of the frequency of pathogenic variants in neonatal HTT.

Genetic approaches based on systematic sequencing and analysis of customized and well-designed panels of genes would further outline the etiology of isolated neonatal HTT. Such studies and long follow-up of patients are needed. Furthermore, the genetic analysis would also permit familial genetic counseling (65).

Before proceeding to molecular analysis or in areas where genetic studies are not available, it seems reasonable to corroborate TSH levels in first-degree relatives of newborns presenting with HTT. According to data in **Table S5**, it can be estimated that more than 50% (17/33) of the first-degree relatives of both biallelic and monoallelic carriers of genetic variants also show increased TSH values.

Since there are no estimates of the frequency of pathogenic variants in neonatal HTT, it is still unknown if the genetic variation is the most common causation of persistent neonatal-onset HTT.

Abnormal thyroid imaging tests were found only in 5 of 28 cases carrying sequence variants in the *TSHR* gene, so it can be speculated that thyroid abnormalities are a different etiology of neonatal HTT (**Table S5**). In this systematic review, the overall frequency of thyroid abnormalities in neonatal HTT was estimated at 27%; however, this percentage decreases to 11% (27/248) after excluding one outlier study (Oren A 2016, **Table S4**) (40).

### What Have We Learned About Management From Follow-Up Studies?

The last consensus about the biochemical criteria used in the decision to start treatment for CH suggests that if the serum TSH concentration is 6–20 mU/L beyond the age of 21 days in a healthy neonate with an FT4 concentration within the age-specific reference interval, either start L-T4 treatment immediately and retest, off-treatment, at a later stage, or withhold treatment but retest 1 to 2 weeks later (3). In this “gray area,” treatment versus observation is based on clinical judgment due to lack of evidence in favor or against treatment. The management of infants with TSH elevations between 6 and 10 mU/L that persist after the first month of life is especially controversial (1).

It is not an easy decision to treat infants based on a single laboratory abnormality. Consistent with the mild nature of neonatal HTT, LT-4 treatment is started at an older age and at a lower dose compared with classic CH (40). The majority of neonates diagnosed with HTT have apparently normal thyroid glands, such that imaging studies do not help in the treatment decisions (66). Undertaking thyroid imaging diagnostic tests should not delay the decision on observation versus treatment beyond 4 weeks of age.

The available evidence is still insufficient to establish whether genetic analyses might represent a helpful diagnostic tool for a tailored management of patients with HTT, and whether it would allow discrimination between conditions that may require L-T4 therapy and follow-up. In any case, at present, genetic studies usually take more than a few weeks, and hence, such studies may not be helpful in treatment decision-making.

It deserves mentioning that most patients with thyroid abnormalities (77/88, **Table S4**) and/or carrying monoallelic or biallelic genetic variants in the *TSHR* gene have received L-T4 treatment (29/33, **Table S5**).

Treatment should be introduced on an individual basis, in discussion with the family. The concerns and anxieties raised in the parents by both repeated examinations and treatment should be carefully considered (57). Withholding treatment and carefully monitoring thyroid function is a rational approach. **Table 1** shows that, in general, conservative management without any medical treatment is applied. Nonetheless, monitoring of thyroid function in infants presenting with HTT is certainly important as some studies have demonstrated cases of patients becoming biochemically hypothyroid, with low T4 and rising TSH levels, requiring treatment with L-T4 (14). Abnormal confirmatory TSH values should be followed up until 2 or 3 years of age, even in patients with an apparent properly adjusted set point for pituitary–thyroid feedback. Unfortunately, few NBS programs routinely follow up detected cases beyond the diagnosis.

After discussing the concern with the parents and paying close attention to avoid overtreatment, some physicians would decide to give L-T4 to infants to maintain both TSH and thyroid hormone levels within the normal range (57). A low dose of L-T4 for HTT infants with frequent monitoring after initiation of treatment seems reasonable. Some authors have reported elevated FT4 levels at some point during the treatment period, and occasionally, L-T4 treatment is stopped due to iatrogenic hyperthyroidism or thyrotoxicosis (8, 25, 67, 68). Therefore, in all those patients for whom L-T4 replacement therapy is started, it is imperative to ensure follow-up until 2 or 3 years of age and to conduct medically supervised trial-off therapy when warranted. This may reduce iatrogenic hyperthyroidism, medical costs, and parental anxiety associated with HTT management (8).

In **Figure 4**, we have presented the approach to the management of neonatal HTT based on the reviewed literature. A matter of concern is whether it is appropriate to resume L-T4 treatment if the TSH levels are slightly elevated following treatment withdrawal. Overall, withdrawal of treatment was judged as unsuccessful, and medication was restarted in 78% of the cases (**Table S7**). Because increased TSH levels seem to be almost always compensated and do not worsen over time, most pediatricians would take the decision to cease treatment and not to resume it despite the presence of SCH beyond 2 or 3 years of age. Children should remain under observation to ensure suitable levels of FT4 throughout childhood.

### Are There Factors Associated With Long-Term Clinical Evolution?

The prediction of the long-term evolution of these infants is still difficult. The evolution of thyroid function cannot be predicted by thyroid imaging tests. The occurrence of abnormal imaging tests was found to be higher in the persistent than in the transient form of HTT. However, the overall frequency of abnormal imaging tests in neonatal HTT is low (**Table S4**).

The clinical spectrum of thyroid dysfunction caused by sequence variants in the *TSHR* gene is wide. However, mildly altered thyroid tests are commonly observed during childhood or adolescence in these patients (**Table S5**).

The variable phenotypic expression can be seen in cases with the same genotype. Mizuno et al. performed a clinical investigation of Japanese patients who had HTT as neonates, in whom a homozygous R450H sequence variant had been demonstrated. They found that although patients may not exhibit obvious hypothyroidism in infancy, resistance to TSH will become apparent with time (29). The elevation of circulating TSH would sometimes represent a compensatory mechanism in the presence of partial refractoriness to TSH action (69). Thyroid hormones are critical for growth and metabolic functions, and thyroid functional requirements increase in certain moments of life such as the neonatal period and puberty. It is always possible that more stringent environmental challenges, such as mild iodine deficiency, can stretch the limited resources of these subjects and manifest as overt hypothyroidism (35).

Follow-up is necessary to characterize a transient or persistent disorder. For children and adolescents who have had neonatal HTT, an overall frequency of SCH of 45% was estimated (**Table S7**).

The significance of SCH during childhood is still a matter of debate. This condition suggests a compensated state of primary thyroid failure requiring increased levels of TSH to maintain normal levels of thyroid hormones. Hashimoto’s thyroiditis is by far the most common cause of SCH. However, in many cases, etiology cannot be identified. Idiopathic SCH has been described in children with neonatal isolated HTT.

Prospective studies with long-term close follow-up of children with neonatal-onset HTT are missing. An Italian group has reexamined a cohort of children with neonatal HTT or false-positive results at NBS. SCH was found in 28 of 44 children at the age of 2–3 years. SCH persisted in 14 of 44 (32%) children at an average age of 8 years; however, most of those children reversed to a normal thyroid function during advanced childhood (5). The reason for this normalization with increasing age is unknown. When environmental factors are auspicious, even partially impaired thyroid function may provide sufficient thyroid hormones, and the child will reach normal TSH values. The authors further suggest that these children might be prone to develop hypothyroidism if environmental conditions will become less favorable at a later stage (5).

### Is There Any Convincing Evidence About the Impact on Cognitive Development in These Children That Supports L-T4 Replacement Therapy?

The most interesting question that remains to be answered is undoubtedly whether L-T4 treatment improves the neurodevelopmental outcome. In general, studies have shown normal cognitive development in children with neonatal-onset HTT; nonetheless, conclusions are limited by small sample size, poor adjustment for confounding factors, or absence of clinic-based diagnostic tools.

Observational studies cannot demonstrate causality. As far as infants are concerned, further neurodevelopmental studies are required, including psycho-intellectual evaluation of treated versus untreated infants. While randomized controlled double-blind trials comparing thyroid hormone treatment and placebo (or observation only) on neurodevelopmental would be the best way to definitively answer the question about the benefit of treatment, those studies are unlikely to be performed for ethical reasons. Until now, the precise neurodevelopmental risks posed by mild disease remain uncertain.

As far as we know, this is the first systematic review on neonatal HTT. Within the limitations of this study, it should be mentioned that, in general, our estimations are derived from data extracted from heterogeneous studies. Between-study variability originates mainly from study design, inclusion criteria, and cutoff values for TSH. The most significant limitation found in the existing published data, as previously discussed, is the lack of consensus in definitions and differentiation between transient and persistent HTT.

According to the reviewed literature, HTT is present in neonates with an estimated incidence of 0.06%. Although it is still unknown which is the most common causation of persistent neonatal HTT, if any, infants suspected of persistent neonatal HTT should be closely followed up until 2 or 3 years of age. Long-term follow-up is also needed to detect moments in life when thyroid functional requirements increase, or environmental conditions become less advantageous. Here, it is tempting to consider whether the condition presenting with circulating TSH values fluctuating above the upper limit of the normal range should be termed as permanent HTT. Perhaps, a distinction should also be made between terms persistent and permanent.

It is not a simple decision to treat these infants. We have presented a flowchart for the management of neonatal HTT based on published literature. Although the optimal approach to this condition is still controversial, this systematic review should provide helpful guidance. Until now, the precise neurodevelopmental consequences caused by mild thyroid disease remain uncertain. Controlled studies are needed to determine whether the impact on cognitive outcome in these children supports L-T4 replacement therapy. Meanwhile, accurate assessment of developmental status, close follow-up of affected individuals, and a future systematic review on this specific topic will provide valuable information to improve disease management.

## Author Contributions

MT designed the study. MT and AC screened articles, determined eligibility, and performed data extraction. MT and AC drafted the final manuscript. All authors contributed to the article and approved the submitted version.

## Funding

This work was supported by ANPCyT (PID-2019-0007).

## Conflict of Interest

The authors declare that the research was conducted in the absence of any commercial or financial relationships that could be construed as a potential conflict of interest.

## Publisher’s Note

All claims expressed in this article are solely those of the authors and do not necessarily represent those of their affiliated organizations, or those of the publisher, the editors and the reviewers. Any product that may be evaluated in this article, or claim that may be made by its manufacturer, is not guaranteed or endorsed by the publisher.

## Abbreviations

CH, congenital hypothyroidism; DUOX2, dual oxidase 2; HTT, hyperthyrotropinemia; NBS, newborn screening; SCH, subclinical hypothyroidism; SNV, single-nucleotide variant; TPO, thyroid peroxidase; TRAbs, TSHR (TSH receptor) antibodies.
