# Supplementary material for: Update on Neonatal Isolated Hyperthyrotropinemia: A Systematic Review
Source: Front Endocrinol (Lausanne). 2021 Aug 18;12:643307. doi: 10.3389/fendo.2021.643307 (PMC8416274; doi:10.3389/fendo.2021.643307)
Supplement: Supplementary file 1 [file DataSheet_1.pdf]

Supplementary Information for

"Update on Neonatal Isolated Hyperthyrotropinemia: A Systematic Review"

Authors: Ana E Chiesa and Mariana L Tellechea

Table		Page
**S1**	List of studies excluded after in deep discussion and agreement between authors	2
**S2**	Iodine status in Neonatal Hyperthyrotropinemia	2
**S3**	Maternal and/or neonatal TRAbs and/or TSHR stimulatory antibodies in Neonatal Hyperthyrotropinemia	3
**S4**	Thyroid morphology and function in Neonatal Hyperthyrotropinemia	4
**S5**	Genetic variants in Neonatal Hyperthyrotropinemia	5
**S6**	TSHR gene variants in Neonatal Hyperthyrotropinemia	9
**S7**	Re-evaluation of thyroid function during infancy or childhood in children with Neonatal Hyperthyrotropinemia	10
**S8**	Bone maturation in Neonatal Hyperthyrotropinemia	11

Table S1: List of studies excluded after in deep discussion and agreement between authors.

ID	Reason
Gruters A 1983 (1)<br>Kung AW 1997 (2)<br>Ordookhani A 2008 (3)<br>Calaciura F 1995 (4)<br>Cuestas E 2015 (5)<br>Garg R 2020 (6)<br>Maruo Y 2016 (7) [patient 4, family 1]<br>Mikelsaar RV 1998 (8)<br>Antonozzi I 1980 (9)	positive at NBS (filter paper, cord blood, dried cord, serum) with normal serum (or filter paper) TSH at reexamination (range 10-90 days)
Köhler B 1996 (10)	wide inclusion criteria: “elevated TSH at NBS or at confirmation of diagnosis on the 14th day of life, and normal T4”
Riaño Galán I 2005 (11)	TSH measured in dried cord blood or dried whole blood collected on postnatal day 2-5 (at NBS) but not further confirmed by serum analysis
Freire C 2010 (12)	
Trumpff C 2016 (13)	
Trumpff C 2015 (14)	
Lain SJ 2016 (15)	

Table S2: Iodine status in Neonatal Hyperthyrotropinemia.

Id	Country	Iodine availability	Condition	Outcome	N cases	Increased	Decreased
Nishiyama S 2004 (16)	Japan	[1]	HTT	neonatal UIC	24	15/24 (>20 µg/dl)	na
Demirel F 2007 (17)	Turkey	[2]	HTT	neonatal UIC	36	na	8/36 (<10 µg/dl)
Rovelli R 2010 (18)	Italy	na	HTT	neonatal UIC	3	0/3	na
Vigone MC 2005 (19)	Italy	[3]	p-HTT	neonatal UIC	1	1/1 (>40 µg/dl)	0/1
Yaman AK 2013 (20)	Turkey	[4]	t-HTT	neonatal UIC	3	3/3 (>20 µg/dl)	0/3 (<10 µg/dl)
Czernichow P 1981 (21)	Belgium	na	t-HTT	neonatal plasma iodide	10	0/10	0/10
Summary estimates					77	19/41	8/50

Footnotes:
Data in column “Abnormal iodine status” is expressed as a ratio of the number of participants with “abnormal” iodine status to the total number of cases.
The Iodine availability column contains information about iodine availability during pregnancy and nursing periods. [1] mothers consumed iodine-rich products (iodine-sufficient area). [2] some mothers restricted (iodized) salt consumption due to hypertensive disorder. [3] mother reported periodic use of iodine-containing collutorium. [4] almost all mothers used iodized salt in an iodine-deficient area.
Abbreviations: p-HTT: persistent HTT, t-HTT: transient HTT, UIC: urinary iodine concentration, na: not available.

Table S3: Maternal and/or neonatal TRAbs and/or TSHR stimulatory antibodies in Neonatal Hyperthyrotropinemia.

Id	Condition	Maternal and/or neonatal TSHR blocking antibodies and/or TSHR stimulatory antibodies	Observations
Evans C 2011 (22)	HTT	2/2 presence of maternal TRAbs	maternal hypothyroidism
Azzopardi P 2010 (23)	HTT	1/1 presence of TRAbs in the infant but not in the mother	maternal hypothyroidism
Tamaki H 1989 (24)	t-HTT	1/1 presence of TBII and TSBAb in both mother and infant	maternal autoimmune thyroiditis
Schwingshandl J 1993 (25)	t-HTT	1/1 presence of TBII and TSBAb in both mother and infant	maternal autoimmune thyroiditis
Rovelli R 2010 (18)	HTT	0/3 presence of neonatal TRAbs	maternal autoimmune thyroiditis

Footnotes:
Abbreviations: TRAbs: TSHR antibodies, TBII: Thyrotrophin-Binding Inhibitor Immunoglobulin, TSBAb: Thyroid-Stimulation Blocking antibody, t-HTT: transient HTT, mo: months, wk: weeks.

Table S4: Thyroid imaging in Neonatal Hyperthyrotropinemia.

	Id	Condition	N cases	L-T4	Imaging tool	Finding	
						absent/hypoplasia/ectopy/ decreased uptake	enlarged/increased uptake
1	Demirel F 2007 (17)	HTT	36	36/36	US	1/36 hypoplasia	0/36
2	Altıncık A 2015 (26)	HTT	10	10/10	US	1/10 hypoplasia	0/10
3	Siklar Z 2002 (27)	HTT	43	0/43	US	1/43 low volume	0/43
4	Alberti L 2002 (28)	HTT	6	1/6	US	1*/6 hypoplasia	0/6
5	Oren A 2016 (29)	HTT	69	69/69	99m-Tc scan	5/69 no uptake, 7/69 ectopy, 18/69 hypoplasia, 2/69 hemiagenesis	24/69 increased uptake
6	de Roux N 1996 (30)	HTT	4	3/4	radioiodine scan	0/4	2**/4 slightly enlarged
7	Rovelli R 2010 (18)	HTT	3	3/3	scan	0/3	0/3
8	Evans C 2011 (22)	HTT	1	1/1	US and 99m-Tc scan	0/1	0/1
9	Narumi S 2009 (31)	p-HTT	3	3/3	US and/or scintigraphy	1/3 mild hypoplasia	0/3
10	Ünüvar T 2013 (32)	p-HTT	20	20/20	US and 99m-Tc scan	4/20 hypoplasia	0/20
11	Zung A 2010 (33)	p-HTT	13	13/13	99m-Tc scan	4/13 decreased uptake, 2/13 no uptake, 2/13 ectopy, 1/13 hemiagenesis	0/13
12	Cody D 2003 (34)	p-HTT	3	0/3	99m-Tc scan	1/3 no uptake in 1 lobe	0/3
13	Aguiar L 2016 (35)	p-HTT	8	8/8	US	1/8 dysgenesis	0/8
14	Nagashima T 2001 (36)	p-HTT	1	1/1	US and 123-I scan	1/1 slight hypoplasia	0/1
15	Lucas-Herald A 2013 (37)	p-HTT	1	1/1	US and 99-Tc scan	1/1 slightly small, no uptake	0/1
16	Vigone MC 2005 (19)	p-HTT	1	1/1	US and 123-I scan	0/1	1/1 increased uptake [at 4 yr]
17	Clifton-Bligh R 1997 (38)	p-HTT	1	1/1	US and isotope scan	0/1 [at 2 yr]	0/1
18	Mizuno H 2009 (39)	p-HTT	5	5/5	US and/or 123-I scan	0/5 [after infancy]	0/5
19	Kumorowicz-Czoch M 2011 (40)	p-HTT	6	6/6	US	0/6	0/6
20	Tomita Y 2003 (41)	p-HTT	14	14/14	123-I scan	0/14	0/14
21	Tyfield LA 1991 (42)	p-HTT	2	0/2	132-I or Tc scans	0/2	0/2
22	Srichomkwun P 2017 (43)	p-HTT	1	1/1, later in life	radioiodine scan	0/1	0/1
23	Sunthornthepvarakul T 1995 (44)	p-HTT	1	1/1	radioiodine scan	0/1	0/1
24	Shibayama K 2005 (45)	p-HTT	1	1/1	123-I scan	0/1	0/1
25	Kotani T 2003 (46)	t-HTT	1	1/1, later in life	US and/or 123-I scan	0/1	1/1 enlarged
26	Aguiar L 2016 (35)	t-HTT	5	5/5	US	0/5	0/5
27	Yaman AK 2013 (20)	t-HTT	3	3/3	US	0/3	0/3
28	Ünüvar T 2013 (32)	t-HTT	17	17/17	US and 99m-Tc scan	0/17	0/17
29	Miyai K 1979 (47)	t-HTT	1	0/1	131-I scan	0/1	0/1
30	Cody D 2003 (34)	t-HTT	2	0/2	99-mTc scan	0/2	0/2
31	Czernichow P 1981 (21)	t-HTT	8	0/8	123-I scan	0/8	0/8
32	Abe K 2015 (48)	t-HTT	1	1/1	US	0/1 [at 5 yr]	0/1 [at 5 yr]

33	Zung A 2010 (33)	t-HTT	13	nav	99m-Tc scan	1/13 abnormal	
Summary estimates		ALL	304	-	-	54/291 ***	28/291 ***
		p-HTT	81	-	-	18/81	1/81
		t-HTT	51	-	-	0/38 ***	1/38 ***

Footnotes:
Data in column “L-T4” is expressed as a ratio of the number of LT4-treated patients to the total number of cases.
Data in columns “absent/hypoplasia/ectopy/decreased uptake” and “enlarged/increased uptake” is expressed as a ratio of the number of participants with “abnormal” imaging tests to the total number of cases.
*** excluding dataset 33 (for dataset 33, the nature of the alteration detected in the thyroid scan was not reported).
The number of patients having abnormal imaging tests receiving L-T4 therapy was estimated from datasets where information about L-T4 treatment was available, dataset 33 was excluded from computation due to evidence was not available. * untreated patients (dataset 4). ** LT4-treated patients (dataset 6).
An additional study, Nishiyama S 2004 (16), was not incorporated into Table S4 for the reason that it is not possible to obtain the exact number of subjects having abnormal imaging tests (see subsection “Thyroid imaging in neonatal HTT”).
Abbreviations: p-HTT: persistent HTT, t-HTT: transient HTT, US: ultrasonography.

Table S5: Genetic variants in Neonatal Hyperthyrotropinemia.

Case	Gene	Genotype			L-T4	Thyroid imaging **	Phenotype later in life	Family background	Ethnicity	Study design
1 (30)	TSHR	co	C41S / F525L	LP/P	no	normal	SCH	no	French	case series
2 (30)	TSHR	co	W546* / C390W	LP/P	yes	slightly enlarged	na	no	French	case series
3 (38)	TSHR	co	W546* / R109Q	LP/P	yes	normal	SCH	no	United Kindom, Italy (unrelated parents)	case report
4 (30)	TSHR	co	Q324* / D410N	LP/P	yes	slightly enlarged	na	yes (sibling)	French	case series
5 (36)	TSHR	co	R450H / G498S	LP/P	yes	slightly hypoplastic	SCH	yes (sibling)	Japan (unrelated parents)	case report, RTSH phenotype
6 (49)	TSHR	co	R450H / V473I	LP/LP	yes	na	na	na	Japan (unrelated parents)	case series, RTSH phenotype
7 (49)	TSHR	co	R450H / R519G	LP/LP	yes	na	na	yes (siblings)	Japan (unrelated parents)	case series, RTSH phenotype
8 (49)	TSHR	co	R450H / R519C	LP/LP	yes	na	na	yes (sibling)	Japan (unrelated parents)	case series, RTSH phenotype
9 (31)	TSHR	co	R450H / D403N	LP/LP	yes (trial-off at 3 yr)	na	SCH	na	Japan	population-based
10 (31)	TSHR	co	R450H / G132R	LP/LP	yes (trial-off at 12 yr)	mildly hypoplasic	SCH	no	Japan	population-based
11 (44)	TSHR	co	I167N / P162A	LP/VUS	yes	normal	SCH	yes (parents and siblings)	Germany-Italy/Czech Republic (unrelated parents)	case report
12 (31)	TSHR	ho	R450H	LP	yes (trial-off at 2 yr)	normal	SCH	na	Japan	population-based
13 (45)	TSHR	ho	R450H	LP	yes	normal	SCH	yes (parents and siblings)	Japan (unrelated parents)	case report
14 (39)	TSHR	ho	R450H	LP	yes	normal	SCH	yes (sibling with hypothyroidism)	Japan (unrelated parents)	cohort, R450H carriers
15 (39)	TSHR	ho	R450H	LP	yes	normal	SCH	na	Japan (unrelated parents)	cohort, R450H carriers
16 (39)	TSHR	ho	R450H	LP	yes	normal	SCH	na	Japan (unrelated parents)	cohort, R450H carriers

17 (39)	TSHR	ho	R450H	LP	yes	normal	SCH	na	Japan (unrelated parents)	cohort, R450H carriers
18 (50)	TSHR	ho	L653V	LP	yes (trial-off at 2 yr)	na	SCH	yes (siblings)	Israel (Arab)	case report, **cons. kindred**
19 (51)	TSHR	ho	A593V	LP	yes	normal	SCH	no	Turkey	case report, **cons. kindred**
20 (30)	TSHR	ho	P162A	VUS	yes	normal	na	yes (sibling)	French (**cons. parents**)	case series
21 (52)	TSHR	he	T655* (delAC)	P	yes	no dysgenesis	na	yes	Italy	case series
22 (28)	TSHR	he	T655* (delAC)	P	yes	normal	na	no	Italy, Belgium	case series
23 (52)	TSHR	he	C41S	P	yes	no dysgenesis	na	yes	Italy	case series
24 (52)	TSHR	he	C41S	P	yes	no dysgenesis	na	yes	Italy	case series
25 (52)	TSHR	he	C41S	P	yes	no dysgenesis	na	yes	Italy	case series
26 (28)	TSHR	he	C41S	P	na	normal	normal (at 8 mo)	yes (father)	Italy, Belgium	case series
27 (37)	TSHR	he	C390F	LP	yes (trial-off at 3 yr)	slightly small, poor uptake	SCH	yes (parents with hypothyroidism)	United Kindom	case report
28 (52)	TSHR	he	P68S	VUS	yes	no dysgenesis	na	no	Italy	case series
29 (31)	TSHR	he	A204V	VUS	no	normal	SCH	no	Japan	population-based
30 (52)	TSHR	he	T607I	VUS	yes	no dysgenesis	na	no	Italy	case series
31 (52)	TSHR	he	P162A	VUS	yes	no dysgenesis	na	no	Italy	case series
32 (52)	TSHR	he	I583T	VUS	no	no dysgenesis	na	yes	Italy	case series
33 (53)	TSHR, TPO	co*	Q90P / P264S, G493S	LP/LP	yes (trial-off at 2 yr)	normal	SCH	yes (father and siblings)	Israel (Arab)	case series, **cons. kindred**
34 (46)	TPO	co	G533C / D574-L575del		no	enlarged (goiter)	normal (at 8 mo)	yes (siblings with NBS+ but normal TSH thereafter)	Japan	case report
35 (19)	DUOX2	co	R842* / R376W		yes (trial-off at 4 yr)	increased uptake	SCH	yes (sibling with hypothyroidism)	Italy (unrelated parents)	case report
36 (43)	DUOX2	co	F966Sfs*29 / G488R		no	normal	SCH, LT-4 started	no	England (unrelated parents)	cohort, RTSH phenotype

37 (48)	DUOX2	co	Y1347C / H678R	yes (trial-off at 5 yr)	normal	normal	yes (mother; siblings with NBS+ but normal TSH in childhood)	Japan (unrelated parents)	case report

Footnotes:
Column Genotype from case 1 to case 33 also contains the ACMG classification/interpretation of each TSHR sequence variant (see Table S5).
Data on L-T4 therapy was extracted - when available - as “yes” or “no”.
** at diagnosis or later in life.
The patient’s country or region was registered as a proxy of ethnicity (otherwise, the author’s region was computed).
Abbreviations: co: compound heterozygous, co*: “combinational heterozygous” (cases carrying variants of two different genes), ho: homozygous, he: heterozygous, TH: thyroid hormones, wk: weeks, NBS: newborn screening, cons.: consanguineous, RTSH: Thyrotropin Resistance, na: not available. SCH: increased TSH and normal/low normal FT4 at the time of L-T4 withdrawal or childhood.

Table S6: TSHR gene variants in Neonatal Hyperthyrotropinemia.

Variant	Classification	Location	Functional Studies
C41S	P	ECD	no expression at cell surface, complete absence of ligand binding, ↓↓ cAMP, dominant-negative action (28,30,54)
P68S	VUS ¥	ECD	↓ expression at cell surface, ↓ cAMP (50,52,54)
Q90P	LP ¥	ECD	↓ cAMP response (53)
R109Q	LP	ECD	↓ TSH binding (38,54)
G132R	LP	ECD	↓ expression at cell surface, ↓ cAMP (31,54)
P162A	VUS ¥	ECD	↓ expression at cell surface, ↓ TSH binding, ↑ cAMP EC50 (30,44,54)
I167N	LP	ECD	↓ expression at cell surface, ↓ TSH binding and cAMP (44,54)
A204V	VUS ¥	ECD	↓ expression at cell surface (31,54)
P264S	LP ¥	ECD	↓ cAMP response (53)
Q324*	P	ECD	↓ expression at cell surface, ↓ TSH binding and cAMP (30,54)
C390F	LP	ECD	(37)
C390W	LP	ECD	↓ expression at cell surface, ↓ TSH binding, ↑ constitutive activity and ↓ cAMP response (30,54,55)
D403N	LP ¥	ECD	↓ expression at cell surface, ↓ TSH binding and cAMP (31,54)
D410N	LP	ECD	= TSH binding,↓ cAMP (30,54)
R450H	LP	TMD, first cytoplasmic loop	= expression at cell surface,↓ TSH binding and cAMP (31,36,45,49,54)
V473I	LP	second TMD	= expression at cell surface,↓ cAMP and IP (49,54)
G498S	P	third TMD	↓ expression at cell surface, ↓ TSH binding and cAMP (36,54)
R519G	LP ¥	second cytoplasmic loop	↓ expression at cell surface, ↓ TSH binding, ↓ cAMP and IP (49,54)
R519C	LP ¥	second cytoplasmic loop	↓ expression at cell surface, ↓ TSH binding, ↓ cAMP and IP (49,54)
F525L	LP	second cytoplasmic loop	↓ expression at cell surface, ↓ TSH binding and cAMP (30,54)
W546*	P	fourth TMD	↓ expression at cell surface, ↓ TSH binding and cAMP (30,38,54)
I583T	VUS ¥	fifth TMD	= expression at cell surface, = cAMP response (52)
A593V	LP ¥	fifth TMD	↓ expression at cell surface, ↓ cAMP (51,54)
T607I	VUS ¥	third cytoplasmic loop	= expression at cell surface, = cAMP response (52)
L653V	LP ¥	third extracellular loop	= expression at cell surface, = TSH binding, ↓ cAMP and IP (50,54)
T655* (delAC)	-	third extracellular loop	truncated receptor, no expression at cell surface (28)

Footnotes:
¥ Variants labeled as VUS according to Varsome were revised and reclassified according to functional studies and data from VEP.
Abbreviations: ECD: extracellular domain, TMD: transmembrane domain, cAMP: cyclic adenosine monophosphate, IP: inositol phosphate.

Table 7: Re-evaluation of thyroid function during infancy or childhood in children with Neonatal Hyperthyrotropinemia.

Table 7.1: Follow-up studies where L-T4 therapy was indicated and re-evaluation was performed after withdrawal of treatment.

Id		L-T4	Thyroid status after withdrawal		Not trialed-off (reason why)
			euthyroid	SCH	
1	Demirel F 2007 (17)	36/36<br>L-T4 discontinuation: 3/36	21/36	12/36<br>(9/12 L-T4 restarted)	0/36
2	Zung A 2010 (33)	40/43<br>L-T4 discontinuation: 3/40	12/40	25/40 (L-T4 restarted)	0/40
3	Aguiar L 2016 (35)	41/76<br>L-T4 discontinuation: 16/34*	0/34*	5/34* (L-T4 restarted)	13/34*
4	Ünüvar T 2013 (32)	37/37<br>L-T4 discontinuation: 10/37	7/37	20/37	0/37
5	Daliva A 2000 (56)	14/14	1/14	12/14 (L-T4 restarted)	1/14 (increased TSH on L-T4)
6	Altıncık A 2015 (26)	19/20	4/16*	3/16* SCH<br>(L-T4 restarted)	9/16* (1/9 dysgenesisgoitre, 8/9 increased TSH on L-T4)
7	Oren A 2013 (57)	80/103	8/31*	6/31* SCH<br>(L-T4 restarted)	17/31*
8	Tomita Y 2003 (41)	14/14	0/14	14/14<br>(0/14 LT-4 restarted)	0/14
9	Kumorowicz-Czoch M 2011 (40)	4/4	0/4	4/4 SCH	0/4
10	Rovelli R 2010 (18)	3/129	3/3	0/3 SCH	0/3
Summary estimates		288/476<br>L-T4 discontinuation: 32/281	56/229	101/229<br>(60/77 LT-4 restarted)	40/229

Footnotes:
Data in column “L-T4” is expressed as a ratio of the number of LT4-treated patients to the total number of cases. L-T4 discontinuation: L-T4 discontinuation before trial-off medication.
Data in columns “thyroid status” is expressed as a ratio of the number of participants with normal or “abnormal” outcomes to the total number of cases. Euthyroid refers to normal TSH or normal thyroid function tests.
* to avoid underestimation of frequencies, the total number of cases treated with L-T4 was adjusted subtracting subjects lost to follow-up and < 3 years old (dataset 3: 7/41 lost to follow-up, dataset 6: 3/19 < 3 yr and dataset 7: 49/80 < 3 yr).
Abbreviations: SCH: subclinical hypothyroidism (increased TSH and normal FT4 and/or T4).

Table 7.2: Other studies where infants with neonatal-onset HTT were followed-up or re-evaluated during childhood.

Id		N cases	L-T4	Reevaluation	
				age (yr)	thyroid status
11	Calaciura F 2002 (55)	23	22/23	1.3-3.7 (at trial-off)	16/23 SCH
12	Miki K 1989 (58)	16	5/16 (2-6 mo after birth)	2-7	2/16 SCH
13	Tyfield LA 1991 (42)	3	0/3	5-6	3/3 SCH
14	Cody D 2003 (34)	8	0/8	3	3/8 SCH,<br>1/8 overt hypothyroidism **
Summary estimates		50	-	1.3-7	24/50 SCH

Footnotes:
Data in column "L-T4" is expressed as a ratio of the number of LT4-treated patients to the total number of cases.
Data in columns "thyroid status" is expressed as a ratio of the number of participants with "abnormal" outcomes to the total number of cases (all others were normal).
** patient becomes hypothyroid on follow-up and L-T4 was started (dataset 14).
Abbreviations: mo: months, yr: years, SCH: subclinical hypothyroidism (increased TSH and normal FT4 and/or T4).

Table S8: Bone maturation in Neonatal Hyperthyrotropinemia.

Id	Condition	N cases	Delayed bone maturation	Age at assessment
Altıncık A 2015 (26)	HTT	7	1/7	na
Siklar Z 2002 (27)	HTT	43	1/43	infancy
Tenenbaum-Rakover Y 2009 (50)	p-HTT	1	0/1	neonatal period
Mizuno H 2009 (39)	p-HTT	4	0/4	17-61 days
Clifton-Bligh R 1997 (38)	p-HTT	1	0/1	na
Nagashima T 2001 (36)	p-HTT	2	0/2	na
Abe K 2015 (48)	t-HTT	1	0/1	neonatal period
Miyai K 1979 (47)	t-HTT	1	0/1	9 mo
Czernichow P 1981 (21)	t-HTT	14	0/14	na
Summary estimates		74	2/74	-

Footnotes:
Data in column "L-T4" is expressed as a ratio of the number of LT4-treated patients to the total number of cases.
Data in column "Delayed bone maturation" is expressed as a ratio of the number of participants with delayed bone maturation to the total number of cases.
Abbreviations: p-HTT: persistent HTT, t-HTT: transient HTT, na: not available.

References

1. Grüters A, L'Allemand D, Heidemann PH, Schürnbrand P. Incidence of iodine contamination in neonatal transient hyperthyrotropinemia. *Eur J Pediatr* (1983) **140**:299–300. doi:10.1007/BF00442668

2. Kung AWC, Lao TT, Low LCK, Pang RWC, Robinson JD. Iodine insufficiency and neonatal hyperthyrotropinaemia in Hong Kong. *Clin Endocrinol (Oxf)* (1997) **46**:315–319. doi:10.1046/j.1365-2265.1997.1310960.x

3. Ordookhani A, Pearce EN, Mirmiran P, Azizi F, Braverman LE. Transient congenital hypothyroidism in an iodine-replete area is not related to parental consanguinity, mode of delivery, goitrogens, iodine exposure, or thyrotropin receptor autoantibodies. *J Endocrinol Invest* (2008) **31**:29–34. doi:10.1007/BF03345563

4. Calaciura F, Mendorla G, Distefano M, Castorina S, Fazio T, Motta RM, Sava L, Delange F, Vigneri R. Childhood IQ measurements in infants with transient congenital hypothyroidism. *Clin Endocrinol (Oxf)* (1995) **43**:473–477. doi:10.1111/j.1365-2265.1995.tb02620.x

5. Cuestas E, Gaido MI, Capra RH. Transient neonatal hyperthyrotropinemia is a risk factor for developing persistent hyperthyrotropinemia in childhood with repercussion on developmental status. *Eur J Endocrinol* (2015) **172**:483–490. doi:10.1530/EJE-13-0907

6. Garg R, Sait H, Jindal A, Juneja M, Gupta S, Thelma B, Kapoor S. Factors Associated with Transient Neonatal Hyperthyrotropinemia. *Indian J Pediatr* (2020) **87**:482–483. doi:10.1007/s12098-019-03095-z

7. Maruo Y, Nagasaki K, Matsui K, Mimura Y, Mori A, Fukami M, Takeuchi Y. Natural course of congenital hypothyroidism by dual oxidase 2 mutations from the neonatal period through puberty. *Eur J Endocrinol* (2016) **174**:453–463. doi:10.1530/EJE-15-0959

8. Mikelsaar R V, Zordania R, Viikmaa M, Kudrjavtseva G. Neonatal screening for congenital hypothyroidism in Estonia. *J Med Screen* (1998) **5**:20–21. doi:10.1136/jms.5.1.20

9. Antonozzi I, Dominici R, Andreoli M, Monaco F. Neonatal screening in Italy for congenital hypothyroidism and metabolic disorders: hyperphenylalaninemia, maple syrup urine disease and

homocystinuria. *J Endocrinol Investig Off J Ital Soc Endocrinol* (1980) **3**:357–363. doi:10.1007/BF03349371

10. Köhler B, Schnabel D, Biebermann H, Gruters A. Transient congenital hypothyroidism and hyperthyrotropinemia: normal thyroid function and physical development at the ages of 6-14 years. *J Clin Endocrinol Metab* (1996) **81**:1563–1567. doi:10.1210/jcem.81.4.8636368

11. Riaño Galán I, Sánchez Martinez P, Pilar Mosteiro Diaz M, Rivas Crespo MF. Psycho-intellectual Development of 3 Year-old Children with Early Gestational Iodine Deficiency. *J Pediatr Endocrinol Metab* (2005) **18**:1265–1272. doi:10.1515/JPEM.2005.18.S1.1265

12. Freire C, Ramos R, Amaya E, Fernández MF, Santiago-Fernández P, Lopez-Espinosa MJ, Arrebola JP, Olea N. Newborn TSH concentration and its association with cognitive development in healthy boys. *Eur J Endocrinol* (2010) **163**:901–909. doi:10.1530/EJE-10-0495

13. Trumpff C, De Schepper J, Vanderfaeillie J, Vercruysse N, Van Oyen H, Moreno-Reyes R, Tafforeau J, Vandevijvere S. Neonatal thyroid-stimulating hormone concentration and psychomotor development at preschool age. *Arch Dis Child* (2016) **101**:1100–1106. doi:10.1136/archdischild-2015-310006

14. Trumpff C, De Schepper J, Vanderfaeillie J, Vercruysse N, Van Oyen H, Moreno-Reyes R, Tafforeau J, Vanderpas J, Vandevijvere S. Thyroid-stimulating hormone (TSH) concentration at birth in Belgian neonates and cognitive development at preschool age. *Nutrients* (2015) **7**:9018–9032. doi:10.3390/nu7115450

15. Lain SJ, Bentley JP, Wiley V, Roberts CL, Jack M, Wilcken B, Nassar N. Association between borderline neonatal thyroid-stimulating hormone concentrations and educational and developmental outcomes: a population-based record-linkage study. *Lancet Diabetes Endocrinol* (2016) **4**:756–765. doi:10.1016/S2213-8587(16)30122-X

16. Nishiyama S, Mikeda T, Okada T, Nakamura K, Kotani T, Hishinuma A. Transient hypothyroidism or persistent hyperthyrotropinemia in neonates born to mothers with excessive iodine intake. *Thyroid* (2004) **14**:1077–1083. doi:10.1089/thy.2004.14.1077

17. Demirel F, Bideci A, Çamurdan MO, Cinaz P. L-thyroxin treatment in infants with

hyperthyrotropinaemia: 4-Year experience. *Int J Clin Pract* (2007) **61**:1333–1336. doi:10.1111/j.1742-1241.2006.00998.x

18. Rovelli R, Vigone MC, Giovanettoni C, Passoni A, Maina L, Corrias A, Corbetta C, Mosca F, Chiumello G, Weber G. Newborn of mothers affected by autoimmune thyroiditis: the importance of thyroid function monitoring in the first months of life. *Ital J Pediatr* (2010) **36**:24. doi:10.1186/1824-7288-36-24

19. Vigone MC, Fugazzola L, Zamproni I, Passoni A, Di Candia S, Chiumello G, Persani L, Weber G. Persistent mild hypothyroidism associated with novel sequence variants of the DUOX2 gene in two siblings. *Hum Mutat* (2005) **26**:395. doi:10.1002/humu.9372

20. Kutlu Yaman A, Demirel F, Ermiş B, Etem Pişkin I. Maternal and neonatal urinary iodine status and its effect on neonatal TSH levels in a mildly iodine-deficient area. *JCRPE J Clin Res Pediatr Endocrinol* (2013) **5**:90–94. doi:10.4274/Jcrpe.997

21. CZERNICHOW P, VANDALEM JL, HENNEN G. Transient Neonatal Hyperthyrotropinemia: A Factitious Syndrome due to the Presence of Heterophilic Antibodies in the Plasma of Infants and Their Mothers. *J Clin Endocrinol Metab* (1981) **53**:387–393. doi:10.1210/jcem-53-2-387

22. Evans C, Gregory JW, Barton J, Bidder C, Gibbs J, Pryce R, Al-Muzaffar I, Ludgate M, Warner J, John R, et al. Transient congenital hypothyroidism due to thyroid-stimulating hormone receptor blocking antibodies: A case series. *Ann Clin Biochem* (2011) **48**:386–390. doi:10.1258/acb.2011.011007

23. Azzopardi P, Forrester M, Ehtisham S. Three siblings with self-resolving congenital hyperthyrotropinaemia secondary to thyrotropin receptor blocking antibodies. *J Paediatr Child Health* (2010) **46**:439–441. doi:10.1111/j.1440-1754.2009.01687.x

24. Tamaki H, Amino N, Aozasa M, Mori M, Iwatani Y, Tachi J, Nose O, Tanizawa O, Miyai K. Effective Method for Prediction of Transient Hypothyroidism in Neonates Born to Mothers with Chronic Thyroiditis. *Am J Perinatol* (1989) **6**:296–303. doi:10.1055/s-2007-999597

25. SCHWINGSHANDL J, DONAGHUE K, LUTTRELL B, COWELL C, WARD P, SILINK M. Transient congenital hypothyroidism due to maternal thyrotrophin binding inhibiting immunoglobulin. *J Paediatr*

Child Health (1993) **29**:315–318. doi:10.1111/j.1440-1754.1993.tb00521.x

26. Altıncık A, Demir K, Çatlı G, Abacı A, Böber E. The Role of Thyrotropin-Releasing Hormone Stimulation Test in Management of Hyperthyrotropinemia in Infants. *J Clin Res Pediatr Endocrinol* (2015) **7**:211–216. doi:10.4274/jcrpe.1985

27. Şiklar Z, Tezer H, Dallar Y, Tanyer G. Borderline congenital hypothyroidism in the neonatal period. *J Pediatr Endocrinol Metab* (2002) **15**:817–821. doi:10.1515/jpem.2002.15.6.817

28. Alberti L, Proverbio MC, Costagliola S, Romoli R, Boldrighini B, Vigone MC, Weber G, Chiumello G, Beck-Peccoz P, Persani L. Germline mutations of TSH receptor gene as cause of nonautoimmune subclinical hypothyroidism. *J Clin Endocrinol Metab* (2002) **87**:2549–2555. doi:10.1210/jcem.87.6.8536

29. Oren A, Wang MK, Brnjac L, Mahmud FH, Palmert MR. Use of Tc-99 m thyroid scans in borderline congenital hypothyroidism. *Clin Endocrinol (Oxf)* (2016) **84**:438–444. doi:10.1111/cen.12807

30. De Roux N, Misrahi M, Brauner R, Houang M, Carel JC, Granier M, Le Bouc Y, Ghinea N, Boumedienne A, Toublanc JE, et al. Four families with loss of function mutations of the thyrotropin receptor. *J Clin Endocrinol Metab* (1996) **81**:4229–4235. doi:10.1210/jc.81.12.4229

31. Narumi S, Muroya K, Abe Y, Yasui M, Asakura Y, Adachi M, Hasegawa T. TSHR mutations as a cause of congenital hypothyroidism in Japan: A population-based genetic epidemiology study. *J Clin Endocrinol Metab* (2009) **94**:1317–1323. doi:10.1210/jc.2008-1767

32. Tolga Ü, Korcan D, Ayhan A, Atilla B, Ece B. The Role of Initial Clinical and Laboratory Findings in Infants With Hyperthyrotropinemia to Predict Transient or Permanent Hypothyroidism. *J Clin Res Pediatr Endocrinol* (2013) **5**:170–173. doi:10.4274/Jcrpe.931

33. Zung A, Tenenbaum-Rakover Y, Barkan S, Hanukoglu A, Hershkovitz E, Pinhas-Hamiel O, Bistritzer T, Zadik Z. Neonatal hyperthyrotropinemia: Population characteristics, diagnosis, management and outcome after cessation of therapy. *Clin Endocrinol (Oxf)* (2010) **72**:264–271. doi:10.1111/j.1365-2265.2009.03634.x

34. Cody D, Kumar Y, Ng SM, Didi M, Smith C. The differing outcomes of hyperthyrotropinaemia. *J Pediatr Endocrinol Metab* (2003) **16**:375–378. doi:10.1515/JPEM.2003.16.3.375

35. Aguiar L, Garb J, Reiter E, Visintainer P, Singh R, Allen H, Tonyushkina K. Can One Predict Resolution of Neonatal Hyperthyrotropinemia? *J Pediatr* (2016) **174**:71-77.e1. doi:10.1016/j.jpeds.2016.04.011

36. Nagashima T, Murakami M, Onigata K, Morimura T, Nagashima K, Mori M, Morikawa A. Novel inactivating missense mutations in the thyrotropin receptor gene in Japanese children with resistance to thyrotropin. *Thyroid* (2001) **11**:551–559. doi:10.1089/105072501750302859

37. Lucas-Herald A, Bradley T, Hermanns P, Jones J, Attaie M, Thompson E, Pohlenz J, Donaldson M. Novel heterozygous thyrotropin receptor mutation presenting with neonatal hyperthyrotropinaemia, mild thyroid hypoplasia and absent uptake on radioisotope scan. *J Pediatr Endocrinol Metab* (2013) **26**:583–586. doi:10.1515/jpem-2012-0308

38. Clifton-Bligh RJ, Gregory JW, Ludgate M, John R, Persani L, Asteria C, Beck-Peccoz P, Chatterjee VKK. Two novel mutations in the thyrotropin (TSH) receptor gene in a child with resistance to TSH. *J Clin Endocrinol Metab* (1997) **82**:1094–1100. doi:10.1210/jc.82.4.1094

39. Mizuno H, Kanda K, Sugiyama Y, Imamine H, Ito T, Kato I, Togari H, Kamoda T, Onigata K. Longitudinal evaluation of patients with a homozygous R450H mutation of the TSH receptor gene. *Horm Res* (2009) **71**:318–323. doi:10.1159/000223415

40. Kumorowicz-Czoch M, Tylek-Lemanska D, Starzyk J. Thyroid dysfunctions in children detected in mass screening for congenital hypothyroidism. *J Pediatr Endocrinol Metab* (2011) **24**:141–145. doi:10.1515/JPEM.2011.080

41. Tomita Y, Ishiguro H, Shinagawa T, Kubota C, Shinohara O. Persistence of mild hyperthyrotropinemia after discontinuation of three-year course of low-dose L-thyroxine therapy in infants with borderline hypothyroidism. *Endocr J* (2003) **50**:379–384. doi:10.1507/endocrj.50.379

42. Tyfield LA, Abusrewil SSA, Jones SR, Savage DCL. Persistent hyperthyrotropinaemia since the neonatal period in clinically euthyroid children. *Eur J Pediatr* (1991) **150**:308–309. doi:10.1007/BF01955927

43. Srichomkwun P, Takamatsu J, Nickerson DA, Bamshad MJ, Chong JX, Refetoff S. DUOX2 Gene Mutation Manifesting as Resistance to Thyrotropin Phenotype. *Thyroid* (2017) **27**:129–131.

doi:10.1089/thy.2016.0469

44. Sunthornthepvarakul T, Gottschalk ME, Hayashi Y, Refetoff S. Resistance to Thyrotropin Caused by Mutations in the Thyrotropin-Receptor Gene. *N Engl J Med* (1995) **332**:155–160. doi:10.1056/NEJM199501193320305

45. Shibayama K, Ohyama Y, Hishinuma A, Yokota Y, Kazahari K, Kazahari M, Ieiri T, Matsuura N. Subclinical hypothyroidism caused by a mutation of the thyrotropin receptor gene. *Pediatr Int* (2005) **47**:105–108. doi:10.1111/j.1442-200x.2005.02020.x

46. Kotani T, Umeki K, Kawano JI, Suganuma T, Hishinuma A, Ieiri T, Harada S. Partial iodide organification defect caused by a novel mutation of the thyroid peroxidase gene in three siblings. *Clin Endocrinol (Oxf)* (2003) **59**:198–206. doi:10.1046/j.1365-2265.2003.01823.x

47. Miyai K, Amino N, Nishi K, Fujie T, Nakatant K, Nose O, Harada T, Yabuuchi H, Doi K, Yamamoto T, et al. Transient infantile hyperthyrotropinaemia: Report of a case. *Arch Dis Child* (1979) **54**:965–967. doi:10.1136/adc.54.12.965

48. Abe K, Narumi S, Suwanai AS, Hamajima T, Hasegawa T. Pseudodominant inheritance in a family with nonautoimmune hypothyroidism due to biallelic DUOX2 mutations. *Clin Endocrinol (Oxf)* (2015) **83**:394–398. doi:10.1111/cen.12622

49. Tsunekawa K, Onigata K, Morimura T, Kasahara T, Nishiyama S, Kamoda T, Mori M, Morikawa A, Murakami M. Identification and functional analysis of novel inactivating thyrotropin receptor mutations in patients with thyrotropin resistance. *Thyroid* (2006) **16**:471–479. doi:10.1089/thy.2006.16.471

50. Tenenbaum-Rakover Y, Grasberger H, Mamanasiri S, Ringkananont U, Montanelli L, Barkoff MS, Dahood AMH, Refetoff S. Loss-of-function mutations in the thyrotropin receptor gene as a major determinant of hyperthyrotropinemia in a consanguineous community. *J Clin Endocrinol Metab* (2009) **94**:1706–1712. doi:10.1210/jc.2008-1938

51. Fricke-Otto S, Pfarr N, Mühlenberg R, Pohlenz J. Mild congenital primary hypothyroidism in a Turkish family caused by a homozygous missense thyrotropin receptor (TSHR) gene mutation (A593 V). *Exp Clin Endocrinol Diabetes* (2005) **113**:582–585. doi:10.1055/s-2005-865914

52. Calebiro D, Gelmini G, Cordella D, Bonomi M, Winkler F, Biebermann H, De Marco A, Marelli F, Libri D V., Antonica F, et al. Frequent TSH receptor genetic alterations with variable signaling impairment in a large series of children with nonautoimmune isolated hyperthyrotropinemia. *J Clin Endocrinol Metab* (2012) **97**:156–160. doi:10.1210/jc.2011-1938

53. Sriphrapradang C, Tenenbaum-Rakover Y, Weiss M, Barkoff MS, Admoni O, Kawthar D, Caltabiano G, Pardo L, Dumitrescu AM, Refetoff S. The coexistence of a novel inactivating mutant thyrotropin receptor allele with two thyroid peroxidase mutations: A genotype-phenotype correlation. *J Clin Endocrinol Metab* (2011) **96**:1001–1006. doi:10.1210/jc.2011-0127

54. Persani L, Calebiro D, Cordella D, Weber G, Gelmini G, Libri D, De Filippis T, Bonomi M. Genetics and phenomics of hypothyroidism due to TSH resistance. *Mol Cell Endocrinol* (2010) **322**:72–82. doi:10.1016/j.mce.2010.01.008

55. Calaciura F, Motta RM, Miscio G, Fichera G, Leonardi D, Carta A, Trischitta V, Tassi V, Sava L, Vigneri R. Subclinical Hypothyroidism in Early Childhood: A Frequent Outcome of Transient Neonatal Hyperthyrotropinemia. *J Clin Endocrinol Metab* (2002) **87**:3209–3214. doi:10.1210/jcem.87.7.8662

56. Daliva AL, Linder B, DiMartino-Nardi J, Saenger P. Three-year follow-up of borderline congenital hypothyroidism. *J Pediatr* (2000) **136**:53–56. doi:10.1016/S0022-3476(00)90049-0

57. Oren A, Wang MK, Brnjac L, Mahmud FH, Palmert MR. Mild neonatal hyperthyrotrophinaemia: 10-year experience suggests the condition is increasingly common but often transient. *Clin Endocrinol (Oxf)* (2013) **79**:832–837. doi:10.1111/cen.12228

58. Miki K, Nose O, Miyai K, Yabuuchi H, Harada T. Transient infantile hyperthyrotrophinaemia. *Arch Dis Child* (1989) **64**:1177–1182. doi:10.1136/adc.64.8.1177
